# Novel dexamethasone-HPMA copolymer conjugate and its potential application in treatment of rheumatoid arthritis

**DOI:** 10.1186/ar2106

**Published:** 2007-01-18

**Authors:** Dong Wang, Scott C Miller, Xin-Ming Liu, Brian Anderson, Xu Sherry Wang, Steven R Goldring

**Affiliations:** 1 Department of Pharmaceutical Sciences, College of Pharmacy, University of Nebraska Medical Center, 986025 Nebraska Medical Center, COP 3026, Omaha, NE 68198-6025, USA; 2 Department of Pharmaceutics and Pharmaceutical Chemistry, University of Utah, 30 South 2000 East, Salt Lake City, UT 84112, USA; 3 Department of Radiology/Radiobiology Division, University of Utah, 729 Arapeen Dr., Salt Lake City, UT 84108, USA; 4 Washington University in St. Louis, 6515 Wydown Blvd., Campus Box 3519, St. Louis, MO 63105, USA; 5 Hospital for Special Surgery, 535 East 70th Street, New York, NY 10021, USA; 6 New England Baptist Bone and Joint Institute, Harvard Institutes of Medicine, 4 Blackfan Circle, Boston, MA 02115, USA

## Abstract

Rheumatoid arthritis (RA) is a chronic autoimmune disease of unknown etiology. Effective treatment of this disorder has been hampered by the lack of availability of agents that selectively target affected joint tissue. We developed a novel pH-sensitive drug delivery system of dexamethasone (Dex) based on an *N*-(2-hydroxypropyl)methacrylamide copolymer (P-Dex) and have shown that the delivery system specifically accumulates in inflamed joints in an animal model of arthritis. We hypothesize that the arthrotropism of the delivery system and the local acidosis-mediated drug release provide superior therapeutic efficacy and potentially reduced side effects in RA treatment. The initial *in vitro *drug-release study confirmed that the Dex release is indeed dependent upon the environmental pH. At pH 5, 37°C, the conjugate shows the highest level of drug release. When administered systemically in an adjuvant-induced arthritis rat model, P-Dex offers superior and longer-lasting anti-inflammatory effects compared with systemically administered free Dex. In addition, greater bone and cartilage preservation was observed with the P-Dex treatment compared with free Dex treatment. Our data indicate that the differential effect of the conjugate is related to its selective accumulation, potential macrophage-mediated retention, and pH-sensitive drug release (extracellular and intracellular) in arthritic joints. This newly developed drug delivery system provides a unique method for selective targeting of glucocorticoids to inflamed joints which may potentially reduce systemic side effects.

## Introduction

Rheumatoid arthritis (RA) is a chronic systemic inflammatory disease that leads to the destruction of diarthrodial joints. Many consider it to be an autoimmune disorder, although the exact cause is unknown. The primary target of the inflammatory process is synovial tissue. The inflamed synovium invades and destroys articular bone and cartilage, leading to significant pain and disability [1-3].

Currently, there is no cure for RA. The most commonly used medications for treatment and management of the disease include nonsteroidal anti-inflammatory drugs, glucocorticoids (GCs), and disease-modifying anti-rheumatic drugs, including the so-called biologic agents that target tumour necrosis factor-alpha and interleukin-1 [1,4]. There is also current emphasis on the early diagnosis and treatment of RA.

Although progress has been made in understanding the molecular mechanisms and identification of novel therapeutic targets for RA, challenges still remain. Most of the available therapies for RA do not have tissue specificity. Therefore, to reach effective drug concentrations in affected joint tissues, high systemic doses of the therapeutic agent must often be administered, which may lead to significant adverse systemic and extra-articular side effects. Reductions in drug doses may attenuate toxicity but may lead to reduced therapeutic efficacy. To overcome this limitation, approaches that specifically target agents to affected joints offer unique promise.

Arthrotropic drug delivery systems may be achieved based on the unique pathophysiological features of RA. Severe synovial membrane inflammation (synovitis) with significant angiogenesis and influx of inflammatory leukocytes is the hallmark of RA [4]. The inflammatory synovial lining, especially the pannus tissue, resembles solid tumors in many ways, including the leaky nature of the associated blood capillaries. This leads to abnormal serum protein infiltration into the synovium and higher protein content in synovial fluid (SF) from patients with RA compared with healthy individuals [5,6]. In solid tumors, similar pathophysiological characteristics lead to the so-called 'enhanced permeability and retention' (EPR) effect for macromolecules [7]. Based on this principle, many colloidal drug delivery systems have been developed for improved cancer chemotherapy [8-11]. There have been relatively few trials using liposome [12], albumin [13], and polyvinylpyrrolidone (PVP) [14] as carriers to deliver anti-rheumatic drugs. More recently, magnetic resonance imaging (MRI) and histological analysis have been used to demonstrate the arthrotropism of *N*-(2-hydroxypropyl)methacrylamide (HPMA) copolymers in an adjuvant-induced arthritis (AIA) rat model [15]. In addition to the arthritic ankle joints, the copolymer showed minor deposition to other inflammatory tissues such as the knee joints and the base of the tail where the adjuvant was given.

Another pathological feature of the rheumatic joint is the presence of low pH. pH values as low as 6.0 have been detected in the SF from RA joints [16-21]. There also appears to be a direct correlation between the acidity of the joint tissues and indices of disease severity [22-24]. The imbalance between increased metabolic activity and insufficient vascular supply, which induces a shift toward anaerobic glycolysis and lactate formation, has been suggested as the cause of the acidosis in RA [17,20]. Similar pathophysiological conditions have been found in solid tumors and exploited to provide a specific drug-releasing mechanism for prodrugs [25,26].

Recently, we designed a novel dexamethasone-HPMA copolymer conjugate (P-Dex) with a pH-sensitive drug-releasing mechanism. Here, we report its synthesis, *in vitro *drug release, and *in vivo *use to treat animals in a rat model of inflammatory arthritis. Our results provide evidence that the therapeutic efficacy of the conjugate is related to its selective accumulation and pH-sensitive drug release (extracellular and intracellular) in arthritic joints. This newly developed drug delivery system provides a unique method for selective targeting of GCs to inflamed joints which may potentially reduce adverse extra-articular side effects.

## Materials and methods

### Materials

HPMA [27], MA-FITC (*N*-methacryloylaminopropyl fluorescein thiourea) [28], *N*-methacryloylglycylglycine (MA-Gly-Gly-OH) [29], and *N, N*-dioctadecyl-*N', N*'-bis(2-hydroxyethyl)propanediamine (LA) [30] were prepared as described previously. Sephadex LH-20 resin was obtained from GE Healthcare (Piscataway, NJ, USA). *N*-(3-Aminopropyl)diethanol amine and carbazic acid *tert*-butyl ester (Boc-NHNH_2_) were obtained from TCI America (Portland, OR, USA). *Mycobacterium tuberculosis *H37Ra (heat-killed, desiccated) was obtained from VWR International (West Chester, PA, USA). Paraffin oil (low viscosity, Bayol F) was obtained from Crescent Chemical Company, Inc. (Islandia, NY, USA). Dexamethasone (Dex) was purchased from Hawkins, Inc. (Minneapolis, MN, USA). If not specified, all other reagents and solvents were purchased from Sigma-Aldrich (St. Louis, MO, USA) and used without further purification.

### Characterization of the synthetic products

The weight average molecular weight (M_w_) and number average molecular weight of copolymers were determined by size exclusion chromatography (SEC) using the ÄKTA fast protein liquid chromatography (FPLC) system (GE Healthcare) equipped with UV and refractive index detectors (KNAUER, Berlin, Germany). SEC measurements were carried out on Superose 12 columns (HR [high-resolution] 10/30) (GE Healthcare) with phosphate-buffered saline (pH 7.3) as the eluent. The average molecular weights of the polymers were calculated using calibrations with poly(HPMA). UV spectra of all tested compounds were obtained on a Cary 400 Bio UV-Vis spectrometer (Varian, Inc., Palo Alto, CA, USA). ^1^H NMR spectra of all synthesized compounds were recorded on a Varian Unity 500-MHz NMR spectrometer (Varian, Inc.). The solvent peak was used for reference (d_6_-dimethyl sulfoxide, 2.49 ppm). Mass spectra of all synthesized compounds were obtained using a Finnigan LCQ DECA mass spectrometer (Thermo Electron Corporation, Waltham, MA, USA) interfaced to an electrospray ionization (ESI) source.

### Synthesis of P-Dex

**Figure 1:**
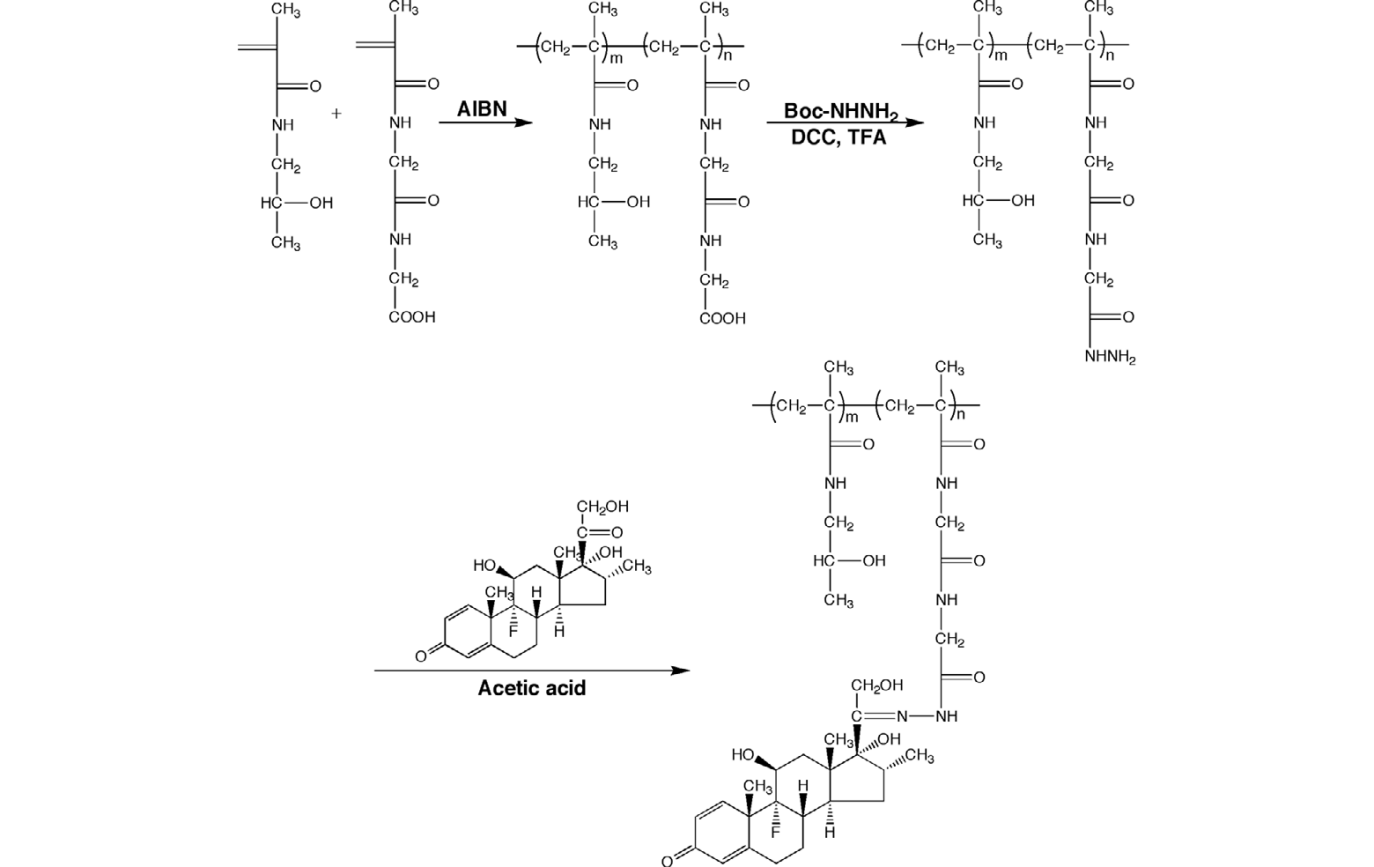
The synthesis of *N*-(2-hydroxypropyl)methacrylamide (HPMA) copolymer-dexamethasone conjugate. Conjugation to dexamethasone may occur at either the 3 or the 20 carbonyl group (an example of the latter is shown). AIBN, 2,2'-azobisisobutyronitrile; Boc-NHNH_2_, carbazic acid *tert*-butyl ester; DCC, *N, N*'-dicyclohexylcarbodiimide; TFA, trifluroacetic acid.

P-Dex was synthesized with the following exemplary procedure (Figure 1). HPMA (1 g, 7 mmol) and MA-Gly-Gly-OH (0.156 g, 0.78 mmol) were copolymerized using AIBN (2,2'-azobisisobutyronitrile) (0.007 g, 0.043 mmol) as initiator. The copolymer (1 g [-COOH] = 0.6 mmol) was then reacted with Boc-NHNH_2 _(1.68 g, 12.6 mmol) using *N*,*N'*-dicyclohexylcarbodiimide (DCC) as the coupling agent. After removal of the resulting dicyclohexylurea and the extra DCC, the Boc (*tert*-butoxycarbonyl) protection in the resulting conjugate was removed by trifluroacetic acid treatment for 2 hours. The resulting polymer was precipitated, dialyzed, and lyophilized to obtain the HPMA copolymer-hydrazide conjugate ([-NHNH_2_] = 4 × 10^-4 ^mol/g). This copolymer (0.75 g) was mixed with an excess of Dex (0.36 g, 9.2 × 10^-4 ^mol) in *N*,*N*-dimethylformamide (1 ml), and one drop of acetic acid was added to catalyze the reaction. It was stirred overnight at room temperature and then purified on an LH-20 column (×2) to remove the unreacted low molecular weight compounds.

### *In vitro *Dex release

P-Dex (1.8 mg/ml) was dissolved in acetate buffer (0.01 M with 0.15 M NaCl, pH 5.0) or phosphate buffer (0.01 M with 0.15 M NaCl, pH 6.0 and pH 7.4) and incubated at three different temperatures (4°C, 25°C, and 37°C). At selected time intervals, the conjugate solution (300 μl) was withdrawn and extracted with ethyl acetate (3 × 400 μl). After Speed Vac^® ^(SC100, Savant Instruments Inc., Holbrook, NY, USA) removal of the solvent, the isolated samples were dissolved in acetonitrile/water (1:1, vol/vol, 600 μl) for high-performance liquid chromatography (HPLC) analysis. An Agilent 1100 HPLC system (Agilent Technologies, Inc., Santa Clara, CA, USA) with a quaternary pump (with degasser), an autosampler, a fluorescence detector, and a diode-array-based UV detector was used for the Dex-release study.

A reverse phase C_18 _(Agilent, 4.6 × 150 mm, 5 μm) was used for the analysis with acetonitrile/water = 1/1 as the mobile phase. Its flow rate was set constant at 0.5 mL/min. The UV detection wavelength was at 240 nm. The sample injection volume was 10 μL for all evaluation. A linear external Dex calibration curve was established in the range of 1 to 150 μg. The calibration was performed with the analysis of each batch of Dex sample. The analysis of each sample was repeated three times. The resulting mean value was converted to the percentage of Dex released.

### Treatment of AIA rats with P-Dex

Male Lewis rats (175 to 200 g) were obtained from Charles River Laboratories, Inc. (Wilmington, MA, USA) and allowed to acclimate for at least 1 week. To induce arthritis, *M. tuberculosis *H37Ra (1 mg) and LA (5 mg) were mixed in paraffin oil (100 μl), sonicated, and injected subcutaneously into the base of the tail [20]. The progression of the joint inflammation was monitored daily. Special care was given to the rats as the inflammation developed to ensure access to water and food. All animal experiments were performed according to a protocol approved by the University of Utah Institutional Animal Care and Use Committee and adhered to *Principles of Laboratory Animal Care *(National Institutes of Health publication 85–23, revised in 1985).

Rats with established arthritis were selected and randomly assigned into three groups (six or seven rats per group). A fourth healthy, untreated group (seven rats) was included as control. On the 13th day after arthritis induction, P-Dex (10 mg/kg, containing 2 mg of Dex/10 mg P-Dex) was injected intravenously into one group of the RA rats. An equivalent total dose of water-soluble free Dex (sodium phosphate) was divided into four aliquots and administered in separate intravenous injections to the second group of rats on days 13, 14, 15, and 16. Saline was similarly given to a third group of rats (controls). The changes in ankle size and body weight during the treatment were monitored.

On day 22, all animals were euthanized and joint tissues were collected. The bone mineral densities (BMDs) of the ankle region (distal tibia to the phalanges of the foot) and the whole femur and lumbar vertebral bodies (fourth and fifth) were measured by peripheral dual x-ray absorptiometry (pDXA). The tissues were fixed in phosphate-buffered formalin for 2 days, and the intact ankle regions were then dehydrated in ascending concentrations of ethanol and embedded undecalcified in methyl methacrylate. Sections of the entire joint were cut with a low-speed saw using diamond-wafering blades. The sections were mounted on plastic slides and ground to approximately 50 μm in thickness, and the surface was stained using a Giemsa stain modified for plastic sections [31]. The ankle joints were assessed for the presence of inflammation and tissue damage. The extent of osteoclastic eroded cancellous bone surface was measured in the calcaneus using an image analysis system (BIOQUANT Image Analysis Corporation, Nashville, TN, USA). The percentage of the cancellous surface undergoing osteoclastic bone resorption as determined by the presence of osteoclasts and resorption pits (eroded surfaced) was calculated.

### Statistical methods

The differences between the groups were first tested by a one-way analysis of variance followed by a Fisher's predicted least-square difference test to determine the significance of individual group comparisons. Differences were considered to be significant at a *p *value of less than 0.05.

## Results

### Synthesis and *in vitro *Dex release from P-Dex

After overnight polymer-analogue reaction between the pendent hydrazide and Dex (acid catalyzed), the conjugate was purified twice using an LH-20 column. A subsequent FPLC analysis of the conjugate indicated that there was no detectable free Dex in the purified copolymer conjugate. The remaining hydrazide in the conjugate was determined using the TNBS (2,4,6-trinitrobenzenesulfonic acid or picrylsulfonic acid) assay [32] and compared with the hydrazide content in the polymer precursor. The reduction of hydrazide content was due to Dex conjugation and the amount of Dex conjugated was determined as 50 mg/g of polymer conjugate. The M_w _of the conjugate was 73 kDa with a polydispersity index of 1.4 and this material was selected for use in the treatment study of the rats. Given that no difference in M_w _was observed for P-Dex and its precursor, the possibility that both carbonyl groups in Dex would react with hydrazide and cause cross-linking of the polymer was minimal.

**Figure 2:**
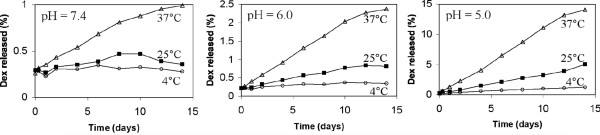
*In vitro *dexamethasone (Dex) release from *N*-(2-hydroxypropyl)methacrylamide (HPMA) copolymer-Dex conjugate at different temperatures and pH levels. *n *= 3, standard deviation is less than 5% of mean value.

For the *in vitro *release study, another batch of P-Dex was synthesized with the Dex content determined to be 106 mg/g polymer conjugate by the TNBS assay and by HPLC after full hydrolysis. As shown in Figure 2, the Dex release rate depends on temperature. When the incubation temperature increased from 4°C to 37°C, the Dex releasing rate was greatly increased. We also confirmed that the release of Dex from P-Dex was indeed pH-dependent. As can be seen in Figure 2, the conjugate demonstrated close to a zero-order release profile during the 14 days at all pH levels tested. The highest drug release occurred at 37°C in the most acidic buffer (pH 5.0), with approximately 14% of the loaded Dex released at the end of 14 days. This amounted to a release rate of approximately 1% of the loaded drug per day. However, all other drug-release pH levels did not yield significant drug release (<5% after 14 days).

### Observation during the treatment of AIA rats

At 13 days after arthritis induction, the ankle size of most of the AIA rats reached a plateau (data not shown). At this time, the hind legs of the affected animals were less mobile and the animals had reduced body weights. Some inflammation was also observed in the front limbs, but it was not as severe as the inflammation observed in the hind limbs. The intravenous administration of free Dex or P-Dex on day 13 led to a rapid suppression of the inflammation. The ankle sizes of both treatment groups were greatly reduced by day 14. Though not quantified, the animals were more mobile and active after the Dex or P-Dex treatments. The effects of P-Dex on the suppression of observable inflammation and mobility and activity lasted for the duration of the entire study (euthanasia on day 22). This was unlike the animals given four daily injections of Dex, in which the inflammation and decreased mobility rapidly returned after the cessation of the Dex injections. By the end of the study on day 22, the animals that had been treated with Dex were indistinguishable from those in the untreated control group.

### BMD assessment

**Figure 3:**
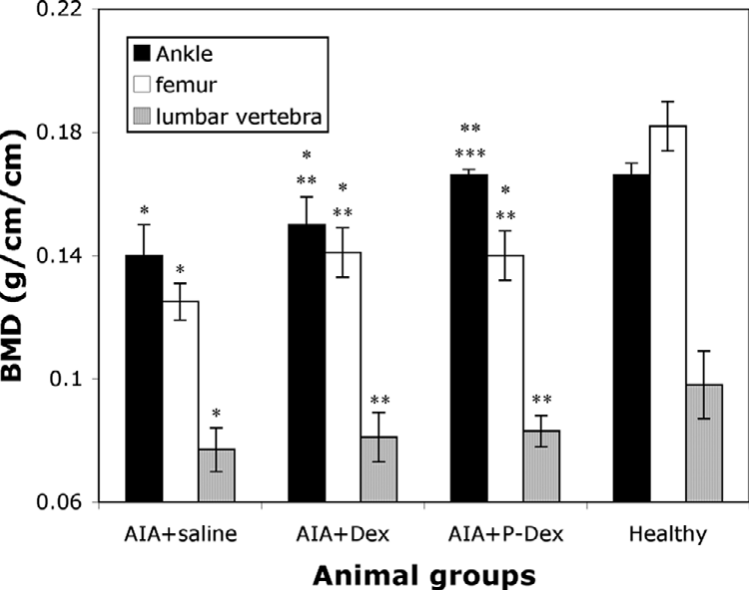
Bone mineral density (BMD) measurement of healthy and adjuvant-induced arthritis (AIA) rats. BMD was evaluated with peripheral dual x-ray absorptiometry at the ankle, femur, and the fourth and fifth lumbar vertebral bodies. *Significantly different from the healthy control group, *p *< 0.05. **Significantly different from the AIA+saline group, *p *< 0.05. ***Significantly different from the AIA+Dex group, *p *< 0.05. Dex, dexamethasone; P-Dex, *N*-(2-hydroxypropyl)methacrylamide (HPMA) copolymer-dexamethasone conjugate.

The measurements of BMD of the ankle region, whole femur, and lumbar vertebral bodies (fourth and fifth) of all animals at the end of the study, as determined by pDXA, are presented in Figure 3. For the ankle region, the AIA+saline group had the lowest BMDs and the values were significantly different from all other groups. The AIA+Dex treatment group had significantly greater BMDs than the untreated AIA+saline group, but the values were significantly less than the healthy controls. The AIA+P-Dex treatment group had BMDs that were significantly greater than those in the AIA+saline and the AIA+Dex-treated groups, and the values were not significantly different from the healthy controls. In the femur, the BMDs of the healthy control group were significantly greater than those of all the other groups. Both the AIA+Dex and AIA+P-Dex treatment groups had significantly greater BMDs than the AIA+saline group. However, the difference between the AIA+Dex and AIA+P-Dex treatment groups did not achieve statistical significance. The BMDs of lumbar vertebral bodies in both the AIA+Dex and AIA+P-Dex treatment groups were significantly greater than those in the AIA+saline group but were not significantly different from each other or from the healthy control. The BMDs of lumbar vertebral bodies in AIA+saline group were significantly less than those in the healthy control group.

### Cancellous bone osteoclastic resorption (eroded) surface measurement

**Figure 4:**
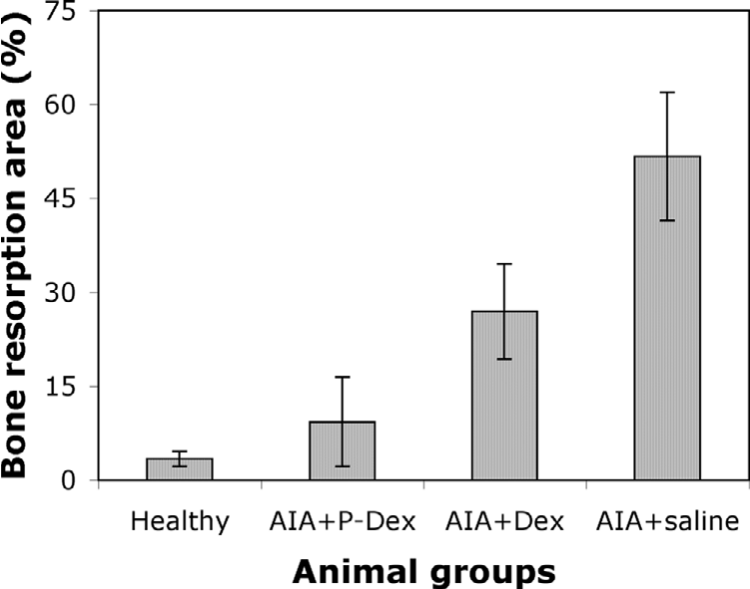
Cancellous bone osteoclast surface measurement in the calcaneus of healthy and adjuvant-induced arthritis (AIA) rats. Significant differences were observed between the following groups: AIA+P-Dex versus AIA+Dex, AIA+P-Dex versus AIA+saline, AIA+Dex versus AIA+saline, AIA+Dex versus healthy, and AIA+saline versus healthy. Differences observed between the AIA+P-Dex versus the healthy group were not significant. Dex, dexamethasone; P-Dex, *N*-(2-hydroxypropyl)methacrylamide (HPMA) copolymer-dexamethasone conjugate.

The percentages of endosteal cancellous bone surfaces undergoing active bone resorption (resorption surface) with the different treatment groups are presented in Figure 4. Almost 50% of the total endosteal surface in the calcaneus of AIA+saline group was undergoing active bone resorption, whereas only 3% of these surfaces were resorbing in the healthy controls. The group treated with Dex (AIA+Dex) had approximately 48% less osteoclast surface than the AIA+saline group, whereas the AIA+P-Dex group had approximately 82% less osteoclast surface. The osteoclast surface in the AIA+P-Dex group was significantly different from both the AIA+saline and AIA+Dex groups, but not from the healthy control group.

### Histological evaluation of ankle joints

**Figure 5:**
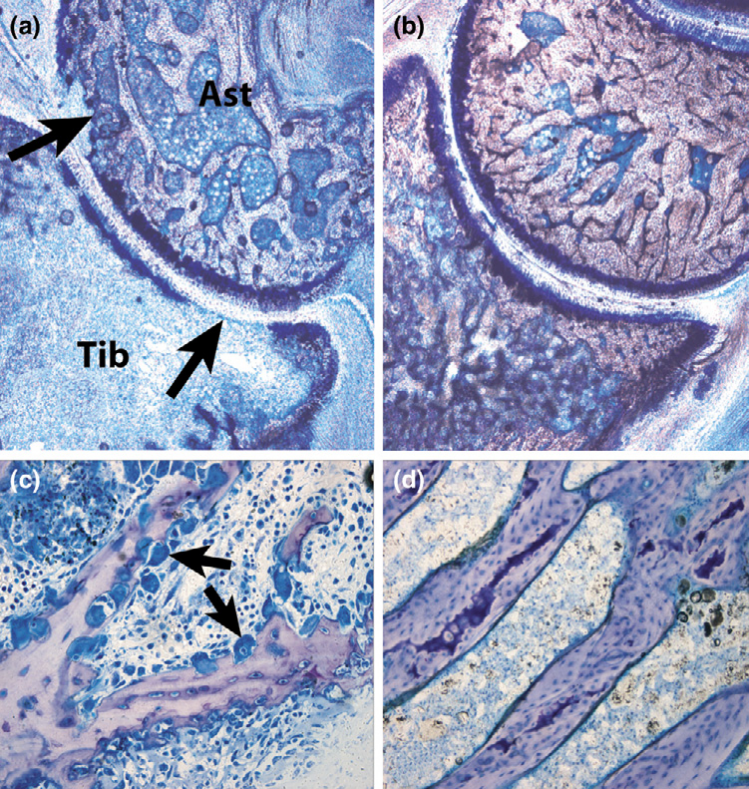
Histological features of the ankle joint from adjuvant-induced arthritis (AIA) rats. **(a) **Tibia (Tib) astrogalus (Ast) joint from rat with saline injection illustrating extensive bone loss, inflammation, and cartilage erosion (arrows). **(b) **Same region from a P-Dex-treated animal showing intact articular cartilage with less subchondral bone erosion. **(c) **Higher-power photomicrograph of cancellous bone from a Dex-treated rat showing extensive osteoclastic (arrows) bone resorption. **(d) **Same region from a P-Dex-treated rat showing much less eroded bone compared to the Dex-treated rat **(c)**. Dex, dexamethasone; P-Dex, *N*-(2-hydroxypropyl)methacrylamide (HPMA) copolymer-dexamethasone conjugate.

Representative histological sections of AIA rats' ankle joints from all treatment groups are shown in Figure 5. Extensive bone loss, inflammation, subchondral bone erosion, and cartilage erosion were evident at the tibia-astrogalus junction in the untreated rats (Figure 5a) compared with the same region in the AIA+P-Dex group (Figure 5b). Cancellous bone surfaces in the untreated controls and the AIA-Dex rats (Figure 5c) were populated with large osteoclasts resorbing bone as indicated by the presence of cells in resorption pits on the bone surfaces. Fewer osteoclasts and less active resorption surfaces were observed in similar cancellous bone regions in the AIA+P-Dex group (Figure 5d).

## Discussion

Colloidal drug delivery systems, including water-soluble polymers, have been used extensively to improve the safety and efficacy of chemotherapeutic treatment for solid tumors [10,11]. The pathophysiological 'EPR' effect is considered as the driving force for their tumor tropic distribution patterns. Drug-releasing mechanisms based on low tissue pH, hypoxia, and unique expression patterns of certain enzymes have been used to enhance the tissue specificity of these delivery systems [11,25,33]. The application of these drug delivery systems to improve the current treatment of RA has not been extensively studied.

PEGylated liposome systems have been used with some success to deliver GCs for the treatment of inflammatory arthritis [12]. As a natural extension, a macromolecular chemotherapeutic agent, albumin-methotrexate conjugate, has also been tested in an arthritic rodent model [13]. Using MRI techniques, we have previously demonstrated that the HPMA copolymer can specifically accumulate and be retained (for 1 to 2 days) in inflamed ankle joints in rats with AIA [15]. Based on these observations, we hypothesized that, due to its preferential deposition to arthritic tissues, HPMA copolymer could selectively deliver a conjugated drug to the inflamed joint tissue while minimizing exposure of extra-articular tissues to the active agent. The benefits of this approach include the ability to increase the therapeutic efficacy by increasing local drug concentration in arthritic joints and the capacity to reduce systemic side effects. Anti-rheumatic drugs with the potential to produce systemic or organ-specific adverse side effects would benefit the most from this approach.

In this proof-of-principle study, we selected Dex as the model compound to be conjugated to the delivery system. Dex, a synthetic GC, is a very potent anti-inflammatory drug that exhibits a rapid therapeutic response. Dex and other GCs are often used in the early phases of RA treatment to relieve symptoms. It has also been reported that these agents can modify the disease progression in patients with RA [34]. However, when used for long-term treatment, GCs are also well known for their adverse side effects, including secondary osteoporosis, muscle weakness and atrophy, suppression of the adrenal gland, increased risk of infection, peptic ulcer disease, and growth retardation [35]. Therefore, a delivery system that could selectively direct GCs to arthritic joints but spare the skeletal and soft tissues would have a significant therapeutic advantage. If the delivery system could enhance the therapeutic index and also reduce the side effects of GCs, it could also be used in conjugation with other anti-rheumatic drugs.

The first challenge for this study was how to conjugate Dex to HPMA copolymer. In a previous report, Dex was conjugated to PVP via its hydroxyl group [14]. However, the ester bond conjugation proved to be too stable to adequately release the conjugated Dex. In the present study, we used a pH-sensitive hydrazone bond to conjugate Dex to the HPMA copolymer side chain. This chemical linkage has been used successfully in conjugating doxorubicin to polymeric drug carriers for improved treatment of solid tumors [25]. As discussed above, acidosis is often associated with inflammatory arthritis. Thus, it was anticipated that the hydrazone bond would be cleaved in the low pH environment of the inflamed joint to release the conjugated Dex, thus enhancing the specificity of the delivery system.

Polymer-analogue reactions were used to conjugate Dex to the HPMA copolymer (Figure 1). As the first step, hydrazide was coupled to the side chain -COOH of HPMA copolymer. After deprotection of the Boc group, Dex was conjugated to the copolymer via a hydrazone bond with acetic acid as the catalyst. The advantage of this synthetic route is its simplicity and ease of purification. However, it is not known which of the two carbonyl groups in the Dex structure was involved in the conjugation. NMR analysis of P-Dex was inconclusive because of the broad peak of the polymeric drug conjugate (data not shown). As evident in this study, batch-to-batch variation of the drug content in the conjugate was significant for the polymer-analogue reaction approach. Synthesis of Dex-containing monomer and its copolymerization with HPMA may resolve the issue. However, the synthesis and especially the purification of the Dex-containing monomer may continue to be difficult.

To validate the pH sensitivity of P-Dex, the conjugate was incubated at three different pH levels in isotonic buffers. As can be seen in Figure 2, the release of Dex from the conjugate was indeed pH-sensitive. At 37°C, Dex release under acidic pH (5.0) is 10 times faster than that released under neutral pH (7.4). The drug-releasing kinetics may be considered as zero-order within the tested time frame, which indicates that the Dex releasing rate is independent of the drug content in P-Dex. Nevertheless, the overall Dex release from the conjugate was only 14% of the original loading after 14 days at 37°C (pH 5.0), or approximately 1% per day. Compared to HPMA copolymer-doxorubicin conjugates, this is rather low [36]. Potentially, the *in vivo *Dex release may be accelerated due to the presence of various proteins that bind hydrophobic drugs.

It is of interest to see that both the free Dex and the P-Dex groups showed immediate relief of inflammation after administration. Because drug-polymer conjugates are not easily recognized by their receptors, their therapeutic activity depends mainly on the amount of free drug released from the conjugate. It usually takes longer for non-targeted polymer-drug conjugates, such as P-Dex, to be endocytosed and incorporated into the lysosomes where the acidic environment would act to release the Dex [37]. The free Dex must then escape from the lysosomal compartment to deliver its anti-inflammatory effect. Therefore, the rapid anti-inflammatory response from the P-Dex observed in this study is best explained by rapid extracellular drug release in the arthritic joint mediated by low extracellular pH. Such a response indirectly confirms the acidosis condition in the arthritic joints of AIA rats. In addition to making general observations of the animals, we measured the change in ankle size with a digital caliper during the treatment. The ankle size data generally agree with the observations discussed above. It is difficult to obtain more specific quantitative measurements for comparison between different groups because of the inconsistency of joint alignment and measurement position.

A previous MRI study suggested that polymeric drug carriers such as HPMA copolymers might be retained in the arthritic joint for at least 1 to 2 days. They were gradually cleared through the urinary tract. However, this cannot explain the observed long-lasting (>9 days) therapeutic effect of P-Dex. One potential explanation is that during its residence in the synovial tissue, colloidal drug carriers (for example, liposome) may be endocytosed by cells such as macrophages [38]. Similarly, if some of the P-Dex was endocytosed, it would remain in the acidic lysosomal compartments and act as a drug depot to gradually release Dex for a prolonged period of time. Considering the relatively slow release of Dex from P-Dex (Figure 2), one may speculate that the amount of free Dex actually needed at the arthritic joint to sustain the suppression of inflammation may be very small.

The ankle joints, compared with other joints, were most affected by the induced arthritis as determined in a previous MRI study and in the present study by histopathology. The advantages of using P-Dex compared with free Dex were also most evident in the ankle joints. The BMD was significantly greater, whereas the relative perimeter of cancellous bone surfaces undergoing active osteoclastic bone resorption was significantly less in the joints from animals treated with one injection of P-Dex compared with four injections of free Dex. In the bones of the ankle joint, the BMD of the P-Dex group was not significantly different from that in healthy controls, indicating that the P-Dex was more effective than free Dex in slowing the bone loss during the disease progression. These differences were not as apparent, however, in the lumbar vertebra and the femur, where both the P-Dex and Dex groups had a greater BMD than the untreated group, but the values were not significantly different from each other. As noted above, the joints in the femur (knee joint) and the lumbar vertebra (intervertebral disks) did not have the same level of inflammation observed in the ankle joints.

Osteoclasts are the cells that resorb bone and are responsible for the bony destruction that accompanies the inflammatory process in RA. The daily treatments with Dex suppressed osteoclastic bone resorption in the ankle bones of the AIA rats compared with the untreated AIA rats, consistent with previous observation. However, osteoclastic bone resorption was further suppressed and was significantly less in the animals given P-Dex compared with free Dex treatment. The fact that the P-Dex was given 9 days prior to the end of the study and that the BMD was preserved in this region indicates that the P-Dex treatment had a sustained effect on limiting osteoclastic bone resorption. There was also less joint destruction in the P-Dex-treated animals compared with those treated with free Dex as confirmed by histological analyses. Further studies will be required to establish time- and dose-related effects of the polymer delivery system on bone and joint metabolism.

In this study, the histology was used to substantiate the efficacy of the treatment with respect to preservation of articular bone structure. pDXA was also used to offer a sensitive measurement of ankle BMD change during the treatment. In future investigations, we will consider using additional imaging modalities such as microcomputed tomography to provide more useful information regarding the preservation of articular bone morphology with this new treatment strategy.

The experiments presented here were not designed for rigorous evaluation of systemic side effects of GC therapy. It would be anticipated that, due to its unique design, this delivery system (which may be viewed as a macromolecular prodrug) would have less systemic toxicity. To activate the prodrug, two conditions must be met: (a) a pathological condition (for example, neovascularization in RA joint) that would allow local enrichment of the prodrug and (b) an acidic environment (for example, RA joint acidosis and lysosomal compartments) that would trigger the release of active Dex from the polymer carrier. Because healthy tissues and organs would lack at least one of these conditions, it would be predicted that this therapy might also reduce the systemic side effects of GC therapy. Exploration of proper animal models is under way to confirm this hypothesis.

## Conclusion

A novel HPMA copolymer-Dex conjugate was designed, synthesized, and tested in an animal model of inflammatory arthritis. The hydrazone bond linking Dex to HPMA copolymer side chains was demonstrated to be cleavable under an acidic pH. When administered systemically, P-Dex proved to offer superior and longer-lasting anti-inflammatory effects compared with free Dex, consistent with its selective accumulation, retention, and pH-sensitive drug release (extracellular and intracellular) in arthritic joints. Greater bone and cartilage preservation was observed with the P-Dex treatment compared with free Dex treatment. This initial study demonstrates that this novel copolymer system may offer therapeutic advantage for the delivery, retention, and release of drugs in the treatment of RA and related forms of inflammatory arthritis.

## Abbreviations

AIA = adjuvant-induced arthritis; BMD = bone mineral density; Boc = *tert*-butoxycarbonyl; Boc-NHNH_2 _= carbazic acid *tert*-butyl ester; DCC = *N*,*N*'-dicyclohexylcarbodiimide; Dex = dexamethasone; EPR = enhanced permeability and retention; FPLC = fast protein liquid chromatography; GC = glucocorticoid; HPLC = high-performance liquid chromatography; HPMA = *N*-(2-hydroxypropyl)methacrylamide; LA = *N, N*-dioctadecyl-*N*', *N*'-bis(2-hydroxyethyl)propanediamine; MA-Gly-Gly-OH = *N*-methacryloylglycylglycine; MRI = magnetic resonance imaging; M_w _= weight average molecular weight; NMR = nuclear magnetic resonance; P-Dex = *N*-(2-hydroxypropyl)methacrylamide copolymer-dexamethasone conjugate; pDXA = peripheral dual x-ray absorptiometry; PVP = polyvinylpyrrolidone; RA = rheumatoid arthritis; SEC = size exclusion chromatography; SF = synovial fluid; TNBS = 2,4,6-trinitrobenzenesulfonic acid.

## Competing interests

DW and SCM are inventors named in a patent application partially related to the content of this manuscript. University of Utah holds the full rights to this patent application. DW and SCM have not received any financial benefit related to this patent application. All other authors declare that they have no competing interests.

## Authors' contributions

DW conceptualized the treatment strategy, designed and synthesized the P-Dex conjugate, and prepared the manuscript. SCM participated in the design of the treatment study, carried out the histology study and data analysis, and participated in the preparation of the manuscript. X-ML participated in the synthesis, characterization, and *in vitro *evaluation of P-Dex. BA participated in the histology study. SXW participated in the synthesis and *in vitro *evaluation of P-Dex. SRG participated in the design of the treatment study, data evaluation, and the preparation of the manuscript. All authors read and approved the final manuscript.
